# Development of the Biomechanical Technologies for the Modeling of Major Segments of the Human Body: Linking the Past with the Present

**DOI:** 10.3390/biology9110399

**Published:** 2020-11-13

**Authors:** Antonio Cicchella

**Affiliations:** Department for Quality of Life Studies, University of Bologna, 40127 Bologna, Italy; antonio.cicchella@unibo.it; Tel.: +39-3393-355-886

**Keywords:** human body segments, body dimensions, biomechanical modeling, anthropometry, body scanning, center of mass determination, body segments inertial parameters, body size

## Abstract

**Simple Summary:**

The procedures of body measurement are as old as the inception of the scientific method. The human being has always had the necessity to shape the environment to its own needs, to care for the body and to improve quality of life. Over the centuries, several methods have been developed to measure body size. With the development of measurement sciences, technological tools as well as computational tools have become increasingly precise. This review paper aims to historically review the development of methods for the measurement of body segments from a biomechanical point of view, highlighting the link with the technologies available today.

**Abstract:**

The knowledge of human body proportions and segmental properties of limbs, head and trunk is of fundamental importance in biomechanical research. Given that many methods are employed, it is important to know which ones are currently available, which data on human body masses, lengths, center of mass (COM) location, weights and moment of inertia (MOI) are available and which methods are most suitable for specific research purposes. Graphical, optical, x-ray and derived techniques, MRI, laser, thermography, has been employed for in-vivo measurement, while direct measurements involve cadaveric studies with dissection and various methods of acquiring shape and size of body segments. The present review examines the literature concerning human body segments’ properties for biomechanical purposes starting with a historical examination. It emerges that data obtained in studies on cadaveric specimens are still accurate in comparison to more recent technologies, whilst technological tools currently available are manifolds, each one with proper advantages and disadvantages. Classical studies were focused mainly on white men, while in recent years, the available data on body segments have been extended to children, women, and other races. Additionally, data on special populations (obese, pregnant women) are starting to appear in the scientific literature.

## 1. Introduction

Human body dimensions have been studied in-depth for a number of reasons: the building of houses and objects, work optimization and workspace design, clinical assessment of walking, in arts, military tasks, aerospace and weapons construction, sports, garment design, ergonomic, and studies on growth. Being aware of body segment lengths and circumference, weight, density, inertial parameters and their own mass displacements is thus important, and the scientific literature reflects this interest. Of special interest is the study of head and trunk and the determination of the body center of mass (COM). COM is a conceptual construct, dating back to the beginning of science. Whole-body COM is paramount for several reasons. The laws of motion are impossible to apply to several objects moving in space, and this is true especially for sports techniques or complex movements because of the constraints imposed by the sport setting. During Olympic or international competitions, it is difficult to directly measure body segment motions with high precision (it is not possible to put markers on the subjects, and joints are covered by garments or sports devices). The COM concept simplifies the calculus and provides a synthetic view of the movement, and the calculation of whole-body kinematics and kinetics. The subject has developed quickly since the availability of fast computation methods. For the biomechanist, it is important to choose the right method considering its reliability, feasibility, ethics and administrative aspects of each method. Additionally, it important to know the origins of the methods; a historical perspective helps in the understanding of how the concept of the measurement of the human body has developed. The measurement and computational methods of choice are important to achieve reliable results. Today, several methods and technological tools are available to the researcher, with their advantages, disadvantages, and proper application. Thus, the aim of this review is to make an updated survey of existing methods for the determination of human body segments inertial parameters. Their origin and development since recent times will be examined, and the pros and cons of each method will be detailed. The availability of new data for special populations will be also reviewed. A historical perspective will be considered, and some methodological issues will be discussed.

## 2. Materials and Methods

**Figure 1:**
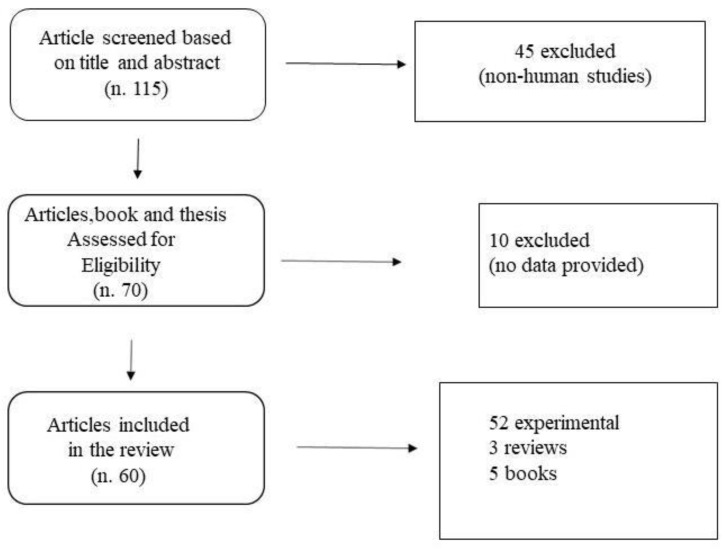
Flow Chart of Literature selection.

An online literature search was performed using PubMed, Google Scholar, Jstor archive, from the inception of the databases to October 2020 with the following keywords used in different combinations: “human body proportions” “computational methods for human segments” “human segments measurement” “human body modelisation” “mathematical methods for human body measurement” “human body proportions and computation”, “segmental biomechanics”. The search strategies were combined, and duplicates were removed by Endnote X7 (Clarivate Analytics, previously Thomson Reuters, Philadelphia, PA, USA) and manually. The databases were queried in a hierarchical way (e.g., first the broader database), starting from Google Scholar. All titles and abstracts were carefully read, and relevant articles were retrieved for review. In addition, the reference lists from both original and review articles retrieved were also reviewed. A total of 115 relevant papers were found. Eligibility criteria limited the search to studies performed on humans, to studies that are related to biomechanics (biomechanical applications), and which examined methods or provided data (experimental studies) on body segment parameter calculation. The studies have to deal with the biomechanical measurement of segments of the human body, describe the technique used, and to be applied in different fields of biomechanics or related to biomechanics (anatomy, ergonomic, orthopedic, bioengineering, mathematical modelization). In total, 60 studies met the eligibility criteria and were included in the review. Of the retrieved papers, 3 were reviews, 5 were reference books, and 52 were experimental. Inclusion criteria were: (i) research conducted with human participants and (ii) related to biomechanics. The exclusion criteria were (i) studies written in languages other than English (ii) animal studies, (iii) congress abstracts. No limits were set concerning the year of publication. The inclusion or exclusion of articles was determined by applying the above criteria on the title and abstract as a first screening and on full texts as a second screening. Case studies were excluded, although the respective references were consulted and integrated into this revision if responding to the above-mentioned criteria (Figure 1).

## 3. Results

### 3.1. Development of Body Measurement Techniques and Methods for Biomechanical Purposes

#### Early Studies

The roots of using optimal human body proportions for design are probably first found in ancient Egypt. The body proportions according to the Neb Aurea (N): 1/N + 1/N2 = 1 (0.618 + 0.382 = 1), lead to a ratio of 1.618. In the Renaissance, we found that this ratio in Leonardo Da Vinci [1] and his Vitruvian Man, and later, the “golden proportion” has been studied by the mathematician Fibonacci in his Series. The “golden ratio or proportion” is observable in the proportions of body organs, such as the ear, the hand, but also in the proportion between limbs of all the body [2]. Alfonso Borrelli [3], in the famous book “De Motu Animalium”, provides the locations of the center of gravity of human and animals (horse, bird), and served as a reference for many scholars of the time. Borrelli employed graphical methods, e.g., depicting figures with their center of mass and studying changes in the center of mass when carrying a weight. These early studies had the purpose of investigating similarities in arts, medicine, architecture, and manufacturing. Volumes, masses, center of mass (COM) location on human body segments, as well as regression equations for the calculation of inertial moments (MOI), are the common output of all the studies dealing with body masses. It soon became clear that the graphical method had several limits, and although human dissection was forbidden, some studies with cadaver dissection began [1]. However, centuries had to pass before the advent of a more rigorous scientific approach.

### 3.2. Eighteenth and Nineteenth Centuries

Distribution of masses in the human body was first calculated directly on two men cadavers of executed prisoners by Harless in 1860 [4]. The cadavers were divided into 18 major segments, with the plane of dissection passing through the pivotal axis of each of the primary joints. The tissues were cut at the center of rotation of each joint that was after disarticulated. The segments then were sutured and weighted, and the volume was calculated from the mass of each segment, assuming a whole-body specific gravity of 1.006. Harless also weighted 44 segments taken from seven corpses. These invasive procedures allowed for the determination of segments weights, weight relative to total body mass, mass, volume and the specific gravity of each segment, location of the centers of masses expressed as the ratio of the distance from the proximal end or joint axis, and the total segment lengths. The complications of these procedures pushed for the development of other methods.

Graphical methods based on the golden ratio were proposed in the architectural domain. Zeising [5] studied human body proportions based on the “golden ratio” for the purpose of adapting buildings to the human body. Additionally, Neufert at the beginning of 1900 [6] employed graphical methods for architectural design purposes. The current development of machine learning for body-size recognition, used to build characters for movies or in security systems, re-vamped golden ratio studies. For example, Abu-Thaie et al. [7] wrote a deep review of the “golden ratio” and developed a new biometric model based on it. We will consider these recent developments further in this paper. The search for standardization never ended in ancient times and continued for centuries. Every epoch is usually accompanied by a new vision, in social life and in science as well. Thus in 1800 and 1900, new standards were proposed for human body proportions and scientific approaches finally bloomed. Braune and Fischer in Germany [8], pioneering the biomechanics of human motion, building apparatus to study the motion. They also performed an autopsy on three middle-aged male suicide specimens. To obtain more reliable results on the center of mass location, they hung the frozen dissected limbs from three axes. Then they calculated the point of intersection of the three planes and marked it on the segment.

Notably in Russia, linked to studies of ergonomics in the heavy “metallurg” (Russian word) industry, Nicholas Bernstein studied the mechanics of the human body for work-related purposes [9] and provided references for body segments of mass, CG and MOI [9]. His famous study about hammer usage is still a classic in ergonomics [9]. However, the work of Bernstein is more connected with the explanations of movements from a control perspective, and he is currently recognized as one of the fathers of motor control studies.

### 3.3. Post World War II

While during World Wars I and II, biomechanical studies were directed toward prosthetics, after WWII, the application of human body proportions studies continued to arise interest for military or space mission purposes. Examples are the classical studies of Dempster et al. [10] and Contini et al. [11], based on the measurement of human cadaver specimens, and aimed at describing the space requirements of a seated operator, his segments lengths and so on. These post-World War II studies were fostered by the space missions and were connected to the new science of space medicine. Reduced gravity posed challenges to the determination of inertial parameters of the human body in space.

Later, experimental methods were developed, including the immersion method [11] for the segmental volumes (from which masses can be obtained), the reaction–change method for estimating segmental masses and locations of centroids, and several techniques applied to cadaveric specimens (quick-release technique, oscillation and suspension of the segments) for determining MOI about segmental joint axes [11]. Immersion methods need the limbs to be immersed in water to measure the volume displacement, and trunk volume is obtained by subtraction from whole-body immersion. Errors can be quite wide due to the choice of segment endpoints. The reaction–change method is based on the use of table boards of different shapes having scales at its vertices. The subject lies on the table, and moves one limb, causing a shift in weight measurement of one scale. These methods are widely used in teaching the concept of COM shifting in biomechanics class and are useful for segmental mass determination of the limbs [11]. This search for methods was fostered by the need of building lower limb prostheses for war veterans and data obtained were helpful for the studies of Inman in prostheses research after World War II [12]. Verne T. Inman, together with Ralston and Todd, was the initiator of biomechanical prostheses research and the determination of MOI on the lower limb became of paramount importance for rehabilitation.

Due to the time consuming of direct measurements, computational approaches were also developed. The Hanavan Model [13] is a mathematical model considering a split human body in 15 different solids, each one having a proper center of mass and proper inertial characteristics, and estimated volumes, weights, and lengths. However, Hanavan assumed equal density for all body segments. To define size and shape, he measured 25 anthropometric parameters. Hands were modeled as spheres, head as an elliptical ellipsoid of revolution, upper (smaller) and lower torso as right elliptical cylinders, and the remaining segments as a frustum of right circular cones. However, this model had some small errors in comparing the obtained total body weight with the summation of all segments weights, which were compensating for distributing the difference between all the segments, albeit not proportionally, thus having a greater effect on the weight error of the limbs.

Chandler and Clauser [14] also provided data from dissections, corrected 40 years later by Hinrich because of some errors in predicting the MOI [15]. They determined body segments′ MOI, calculating them using the pendulum methods, using frozen specimens of 14 male elder cadavers, obtaining more reliable results compared to living subjects’ models. They provided regression equations for each body segment on the 3-principal spatial axis. Their studies were very accurate, and they even accurately measured the local gravitational constant. Hatze [16] provided a model of high computational complexity which required the anthropometric measurement of 17 body segments on living subjects as input and required about 80 min for each subject. Using direct inputs of the subject′s anthropometry, he provided a method that can be tailored to a single subject. The Hatze model is quite complex as it considers body segments as made of slices of 20 mm for which area, mass, COM, and dimensions were computed.

### 3.4. Modern and Contemporary Times

Only with the availability of computerized gamma-ray scanning methods was it possible to calculate, on living humans, body segment dimension (lengths, volumes, and MOI) precisely, without dissection. In the present, there are some concerns about the gamma irradiation of young human subjects, but at the time, these methods seemed ethical and effective. Zatsiorsky and Seyulianov [17] employed gamma-ray scanning to determine body segments inertial parameters, volumes and densities in young, healthy men. These data, like the previous, refer to Caucasian men. Only relatively recently inertial parameters have been published, in relation to different genders and races [18,19], and norms for Asian men were also provided [20,21,22]. There have been attempts to profile a single state population or special small populations, for example, a study on Bulgarian [23] or Indonesian population which showed very different distributions in dimensions, COM location and inertial parameters of these populations in comparison to Caucasian [24]. In particular, feet and hands showed to be longer in Indonesian men relative to Caucasian men. This latter study also provided measurements for the elderly and children. The affordability of X-ray, MRI, CT scan, double beam low dose X-ray absorptiometry (DEXA) [24,25], and 3D infrared scanning [26,27], allows for the screening of large cohorts, and for the personalized screening of body sizes, making population studies feasible. The data structures provided by these methods are quite different. X-ray is used for images of joint center locations as MRI and CT scans, while these two last methods allow also for tissue discrimination, and thus for density calculation, using complex calculations methods. To employ these techniques, a multidisciplinary team is needed. 3D infrared scanning has the advantage of not being irradiating. A problem of possible irradiation remains, even with low doses, for DEXA methods, and the measurements are difficult to replicate due to the subject positioning. 2D biplanar X-ray, resulting in 3D images, is a cheaper method in comparison to MRI, albeit more time consuming and irradiating [28]. Recently, 3D body scanners with optical double triangulation have been proposed as a suitable method for acquiring volumes with a small measurement error of ±1 mm [29]. The subjects are scanned with a surface laser beam, volumes are calculated by light refraction, and COM identified. Irradiating methods have an ethical justification for clinical purposes. All these methods have significant differences, making comparability between different studies difficult [30] and they require an understanding of image processing algorithms. We will consider the issue of variability when examining the literature on single body segments. In addition to the variability between the different methods, there are sources of errors coming from the subject of measurement itself: the human body is not stable over time, and even over a short period of time, due to fluid losses, nutrition and changes in muscular stiffness. This concept of variability is emerging in the scientific literature. For example, during the impact after a jump, localization of segments COM changes up to 17% due to the soft tissue displacement [31].

The study of body segments also attracts new interest today due to the development of additive manufacturing in the industry (e.g., 3D printing) [32] for example, allowing for high customizable limb prostheses. According to Rao et al. [33], the choice of the model for body segment parameter estimation has a strong influence on error results ranging from 9.73% up to 60% for joint kinetics calculation, using the inverse dynamics approach. After reviewing and statistically testing the most used models of the human body for joint kinetics calculations, by using the inverse dynamics approach, they concluded that the Seyulianov–Zatsiorky model obtained with gamma radiation was the most accurate [33]. The need for data on women has fostered by the evidence that body segments inertial parameters are significantly different between men and women in large samples, for example in the study of Challis et al. on 1756 males and 2208 females of different ages [34].

Some body segments deserve special consideration due to their relevance, and because the methods used for their calculation are more complex and informative on the developments of technologies. Studies about the head biomechanical parameters are particularly relevant for understanding trauma, while the trunk segment, being the largest in the body, contributes heavily to whole-body kinematics and kinetics. Displacements of internal organs of the trunk also affect the inertial parameters of the whole body.

#### 3.4.1. Head Segment

The mass and inertial characteristics of the head are of special relevance for injury prevention, mechanics of the impact, the search for brain lesions and evaluation of pre-peri and post-surgery interventions on the brain. Before the availability of accurate CT scan techniques (and mixed CT/MRI techniques) and of fast and accurate software for FEM (finite analysis modeling), these studies were performed on human specimens of head employing destructive trials. The values for head mass found by Yoganandan [35] on nine specimens were similar to the values found by Rousch on four specimens [36], of 4.07 ± 0.0077 kg. Yoganandan [35] reported the data from the literature summarized in Table 1.

Computational models of the head underwent tremendous advances with the increasing computing possibilities [37]. The propagation of shock waves in the skull can be modeled with a certain accuracy knowing the head biomechanical characteristics, using FEM mathematical models. Finite Element Models of the head, fed with CT scan data, allows for the detailed modelization of bones and soft tissue, and enable the very precise estimations of many biomechanical parameters, such as densities, and even joints and muscle actions. The density of the human head was computed giving a density of 1900 kg/m^3^ for the skull, and an almost double 3300 kg/m^3^ for the jawbone [37]. University of Michigan Visible Human Project [38] provides data for volume estimation of a human male body, head and limbs, publicly available. These data can be also useful for the construction of FEM computational models of human limbs. Age and sex of the subjects also affect these calculations, and special population models have also been proposed. Pregnant women underwent a rapid change in body mass distribution [39] with important consequences on back pain. Children, with a more pronounced head mass, have a different distribution, and undergo rapid and uneven changes in segmental development and thus deserve dedicated studies, considering the growth [40].

#### 3.4.2. Trunk Segment

The human trunk, being the largest mass in the body (41.6% of total body mass according to Pearsal et al. [41]), contributes largely to the whole body CG, and deserved special attention in the scientific literature. Many biomechanical models of the human trunk have been proposed, and show a considerable range of error ranging from up to 50% whereas the best ones are in the range of 6% [42]. They found a whole trunk mass ranging from 27 ± 2.52 to 30.86 ± 2.10 kg for females and from 35.90 ± 6.07 to 40.41 ± 5.61 kg for male replicating several different formulas on a cohort of 25 adult males and 25 adult females. The trunk was normally modelized as one or two segments [13], and there have been recent attempts to a more detailed modelization using a four-compartment model: shoulder girdle, thorax, abdomen, and pelvis [43]. The trunk is composed more than other parts of the body, like moving organs. We know that the inertial properties of the lower limb change during an impact, with 17% of leg mass shifts toward the upper part of the limb [31]. We can hypothesize a larger change in the trunk due to internal organ displacements during impact, although no experimental data exists on this point. A model accounting for trunk impact should consider the stiffness and damping ratio for each internal organ. The trunk biomechanical modelization is useful for sports such as rhythmic gymnastics, high jump, gymnastic, and all the movements where a back arch is required or when contact injuries are frequent, such as in American Football. The same study [43] shows that in young people, the lower limb accounts for 37% of total body mass, compared to the 32% found by Zatsiorsky and Seyulianov [17]. This difference can be due to the fact that Zatsiorsky and Seyulianov did not use standard endpoints for segments, and in fact, their data were later adjusted for corrections [44]. CT scan of the trunk shows that trunk CM is 2 forward to L1/L2 while transversal vertebral CM was up to 5 cm forward of vertebral centroids in the lower thoracic region. Trunk modelization is also of interest for not widely practiced sports [45]: the human trunk has been modeled as a two-segment mass in the study of the high jump Fosbury flop technique, which requires to pass the bar performing a back arch [45]. Such a model allowed for the exact determination of the clearance, e.g., the distance between the back and bar at the maximum high of the center of gravity [45].

#### 3.4.3. Localization of the Whole Body Center of Mass

**Figure 2:**
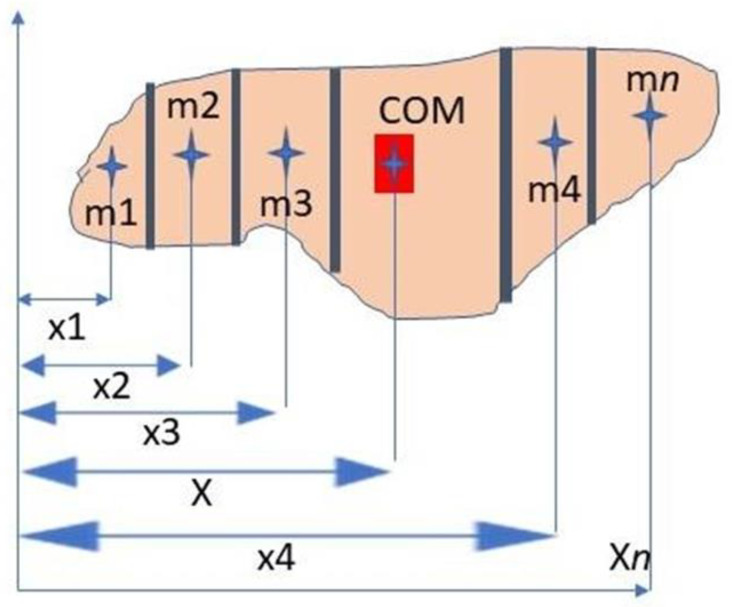
Localization of the center of mass (COM) off an object by means of the segmental method.

Segmentation of the human body allows ultimately for the precise localization of the body center of mass (COM). The concept of COM was introduced by Archimedes [46] together with the graphical method in the static body and has been applied in the Renaissance to study the human body [3]. The fundamental book of Assis (2010) [46] is of paramount importance in understanding how the concept was developed by Archimedes, using practical experiments based on rigid body geometry. COM is an important concept, because it allows for stability during rest and motion, posture and whole-body kinematics, and explains environmental constraints faced by the human body and a number of work-related tasks, for example, carrying loads in military operations [47]. A classical method for whole-body COM is the determination on a bidimensional plane using the individual segments COM. This method is known as the segmental method [48,49] (Figure 2) and is a paradigm to understand how graphical methods work.

(1) $$x\;1/M=\overset{n}{\underset{i}{\sum }}\;m1-x1$$

Equation (1). *x* is the horizontal location of the total body center of mass, *n* is the total number of segments, *m*1 is the mass of the *i*th segment, *M* is the total body mass, and *x*1 is the horizontal location of the *i*th segment center of mass [48,49].

This formula does not consider the different densities of each segment’s zone, which can change dramatically due to the different tissue’s distribution inside a body segment. The method refers to static bodies, and does not consider the displacements of masses inside body segments that happen during motion. Other methods to calculate whole-body COM are suspension methods of cadaver specimens [10,14], shifts in weight distributions using multiple scales [50], digital modeling [51]. Among digital methods, stereophotogrammetry [52,53] or motion analysis can be used [54]. Comparing these methods, and using rigid bodies, such as bricks (as reference measurement standards), the scales methods gave the best repeatability results (within 1.49 mm), followed by digital modeling (within 2.39 mm), and suspension (within 38.5 mm) [54]. Surface laser scanning can also be employed [55] to morph a whole body or to obtain a mean model from a large sample, by means of calculus. Complex human motor behaviors, such as sports, required mathematical modelization of the whole-body COM, allowing for forward kinematics, for example, in platform diving [56]. An avatar, containing all the characteristics of the real diver, can be animated with forward kinematics, to study performance and to identify movements that are possible to perform, due to physical constraints, during competition [56]. This approach is especially helpful in motor performances that cannot be repeated many times or that can be risky for the performer. Image processing is increasingly used in determining whole-body COM because of its minimal invasive characteristics and, recently, for monitoring the movement of people for security reasons, for example in airports, using body shape and movement as biometric tools [7]. Working on images taken by simple cameras (smartphone apps) to calculate COM has the advantages of being fast, cheap, and thus allows the calculation of COM on large samples in reasonable times, albeit the precision can be low.

The method of choice is always a trade-off between precision, reliability and time/costs. Several optical methods have been proposed [57,58] but they have the limits and the lower accuracy (in comparison to direct measurement methods) of real body images (garments which cover the body, localization of body points at distance). To overcome these limitations, recently, artificial intelligence methods with machine learning approaches have been proposed. These systems are built on the capacity of the algorithm to extract accurately segments’ centroids and thus calculate whole-body COM from dressed people in normal environments, learning from the system previous estimation errors, and thus improving accuracy by repeated measurements [58]. However, the current level of accuracy is far from that needed in clinical applications, where precision of a few mm is needed. Inertial sensors can be used in adverse environments such as downhill ski tracks [59] where optical methods are difficult to implement. Repeatability of inertial sensors in this experimental setup is difficult to assess, due to the inner characteristics of the variability of the task itself. However, the desired level of accuracy depends on the purpose of the measurement. Sensors for training monitoring in alpine skiing, albeit not so precise as other methods in estimating COM, can have an acceptable margin of error in field use [59]. An important assumption to determine the COM optically is to know the body density in advance. Optical methods, specifically the ones that provide information on the temperature of an object (tele-thermography), can be used to estimate density by mathematical procedures of neural networking, and used in parallel with shape estimation and limbs centroid calculations, to allow the determination of the center of mass [60].

## 4. Conclusions

The objective of this review was to analyze currently available methods for major body segment determination in biomechanics following a developmental perspective of the techniques and its applications. Up to a few years ago, no data on women, children and other ethnicities than Caucasians were available. In recent years, special populations have been investigated for body segments parameters determination (pregnant women, obese, children, ethnic groups). Several different methods are available today. Early methods employed graphical approaches for single limb and whole-body center of mass and direct cadaver measurement of body segments inertial properties. Head and Trunk deserved special attention in the scientific literature. Head is particularly important because of safety issues, and trunk because it is the largest part of the body, with moving internal organs, thus posing a challenge on the calculation methodologies. Graphical methods, with many variants, are still used because they are inexpensive and non-invasive. Graphical methods have been further developed with the advent of imaging methods, using one or more cameras to identify the COM in the 3D space. More precise determination of limbs and body COMs on images can be achieved with X-ray, 3D X-ray, low dose X-ray (DEXA) and CT scan, but these methods imply the use of radiation. Radiation techniques have been extensively employed in the past but are less used today because of ethical reasons and changes in the ethical requirements of granting institutions. However, data gathered with X-ray and similar methods can still be obtained from ill people who must undergo such investigations for health reasons. MRI is a non-irradiating method, but is not always tolerated by subjects, because it can cause claustrophobia (especially in children) and it is still costly to use on large samples. It must be specified that open MRI machines are becoming more common. Motion analysis systems, inertial sensors, tele-thermography and surface laser scanning have been proposed as reliable methods to measure body segments, with different applications on target populations. Further developments of optical methods include automatic shape recognition and limb weight estimation using density equations and machine learning. When the subjects are in motion, further complications arise due to mass shift, especially in fast movements with sudden stops and go. From a graphical approach, methods have developed toward computation with the parallel development of hardware technologies. The Caucasian centered approach of the early times has developed into a more comprehensive view, encompassing different ethnicities and different human conditions (maternity, growth, obesity). These basic studies are fundamental for building more complex biomechanical analysis. Today, the biomechanist can choose among different methods and available tools to assess the body characteristics of their subjects, allowing for in-depth analysis and more accurate results. The application of new techniques such as machine learning to the field of human body modeling is a promising approach to find better solutions to formerly unsolved problems.

### Final Considerations

This review examined the development of different methods for the measurement of human body segments and for the determination of their mechanical characteristics. Each method presents its own points of strength and weakness. It is shown that precision and reliability normally increase with more elaborated methods, albeit direct measurements on cadavers are still in use for clinical reasons (e.g., endoprostheses design). However, there are ethical (e.g., radiation) and administrative concerns related to the use of more sophisticated methods. CT scans, for example, are still expensive, and not affordable by any laboratory. Thus, the studies which employ the more complex technologies suffer the problem of replication. More simple methods, such as the graphical methods, being more affordable, produced a larger body of knowledge because are easier replicable. The measurement of human body segments, once confined to the study of anatomy, has spread to many different fields of science. Understanding the historic development of the techniques provides a background to the researcher about which method is suitable for a specific purpose. The needs of sport sciences are, for example, quite far from the precision needed for endoprostheses implants. Each method is suitable for a given sample of the population (e.g., children, adults, athletes, ill people) considering the pros and cons. The field is still in rapid development and with the advancement of informatics (e.g., artificial intelligence and machine learning), new methods are rapidly being available at more affordable costs. We can observe a trend toward simplification (e.g., artificial intelligence approach) and more user-friendly methods, which can be used also by non-specialized personnel. Additionally, a trend toward a higher precision of measurement is observable. Today, human morphometrics is a sound body of knowledge that represents a candidate to be a specific trans-disciplinary field of science.

## Figures and Tables

**Table 1:** Mean ± standard deviation of male head mass measured on human specimens. Due to the difficulty of the technique, the different dissection methods, the age span (19 to 70) the results are quite homogeneous. Only nine female specimens were analyzed in literature (seven in Plaga et al.’ and two in Beier et al.’ studies). Modified from [35].

Author	Years	Number of Subjects	Wight (kg) Mean and SD
Harless	1857	2	4.15 ± 0.57
Braune and Fischer	1889	3	4.40 ± 0.80
Fischer	1906	1	3.88
Mertz	1967	3	3.49 ± 0.90
Clauser et al.	1969	13	4.73 ± 0.32
Hodgson et al.	1970	13	3.98 ± 0.53
Hodgson and Thomas	1971	37	4.72 ± 0.78
Walker et al.	1973	19	4.38 ± 0.59
Becker	1972	6	3.88 ± 0.47
Chandler et al.	1975	6	3.99 ± 0.53
Beier et al.	1980	19	4.32 ± 0.40
Albery	2002	1	3.17
Plaga et al.	2005	8	3.66 ± 0.58
Rousch	2010	4	4.07 ± 0.077
Dempester (head and neck)	1955	9	4.60 ± 0.60
